# The social justice dilemma of liability allocation in medical damage: a qualitative analysis based on case judgments

**DOI:** 10.3389/fpubh.2026.1879273

**Published:** 2026-09-01

**Authors:** Yilin Hu

**Affiliations:** School of Law, Renmin University of China, Beijing, China

**Keywords:** causative potency theory, compensation scope, interest balance, medical neglect, medical risk governance

## Abstract

Currently, medical liability regimes in numerous jurisdictions face common dilemmas and challenges linked to social justice. Issues such as excessive medical litigation, the pervasive practice of defensive medicine, and escalating doctor–patient conflicts have significantly negatively affected the operations of global healthcare systems, progressively eroding the foundation of social justice. This has prompted legislative bodies to reevaluate and reform existing medical policies and the structural framework of medical liability systems. The definition and allocation of medical liability require reexamination to prevent liability rules from disproportionately favoring either patients or physicians, thereby advancing the social goals of establishing a “doctor–patient community” and realizing “doctor–patient justice.” The factual causation linkage between medical negligence and patient harm constitutes the core element and “control valve” for the establishment of medical liability, and this area requires continuous advancement in both theoretical research and practical application. This study adopts qualitative case analysis and interpretive theoretical methods as the primary approaches to investigate value conflicts, institutional deficiencies, and context-dependent practical significance embedded in typical medical liability cases. Comparative legal methodology is further employed to explore the root causes, impacts, reform paths and theoretical innovations underlying the alienation of medical liability. This article argues that within the framework of social justice, the “gap-filling principle” derived from tort law theory is insufficient to resolve entrenched doctor–patient conflicts, and the theoretical orientation needs to shift from an exclusive focus on damage compensation to the integration of multiple normative objectives. Proportional causation theory, constructed on the basis of the minimum likelihood probability principle, and the corresponding proportional liability provide groundbreaking insights for resolving the dilemma of determining factual causation standards in medical liability cases. This theory can be integrated with proportional liability under the causative potency doctrine, forming a structurally layered synergistic mechanism to reconstruct the proportional compensation liability for medical damage cases at the level of legal causation. The core solution to the social justice dilemma lies in establishing a dynamic and systematic medical liability regime that (i) clarifies the hierarchical relationship between patient damage remedies, medical professional freedom and social costs; (ii) strikes a balance among patient safety, medical innovation and liability accountability; (iii) realizes risk socialization through mandatory medical liability insurance; and (iv) explores diversified social risk-sharing and healthcare security mechanisms. Ultimately, this approach facilitates the coexistence and mutual promotion of interests among medical institutions, patients, the medical industry and the public.

## Introduction

Medical damage refers to the injury suffered by patients caused by the negligence of hospitals or medical practitioners during diagnosis and treatment. This is a prevalent adverse event in clinical practice worldwide. Any patient receiving medical services may be injured by adverse medical events, which generate substantial social costs and public health burdens and represent a typical source of social conflict. Safeguarding patient safety is not only a core public health mission but also a global priority and shared challenge, and it is a key enabling factor for achieving the United Nations Sustainable Development Goals. In April 2024, the World Health Organization released its first Charter of Patient Safety Rights, which states that “Patient safety speaks to the first, fundamental principle of health care—Do no harm” (1) and calls on stakeholders to formulate relevant laws and policies and advance global cooperation to guarantee patient safety. The harm risks faced by patients have exposed a series of deficiencies in current regulatory frameworks, and issues pertaining to medical liability and disputes have garnered extensive academic and public attention. Harm to patients arising from medical practices is often multifactorial. Constrained by the current limits of medical development, the pathogenic mechanisms of many diseases remain unclear. Owing to the complexity and uncertainty of etiologies, the diversity of available treatment options, and urgent clinical time constraints, clinicians may occasionally exhibit predictable irrational practice behavior, which ultimately causes primary harm to patients’ health and physical integrity (2). The complexity and professional specialization inherent in medical malpractice litigation give rise to high operational costs and systemic inefficiency. A growing volume of claims has driven up medical liability insurance premiums for hospitals and physicians, alongside a rise in the utilization of unnecessary medical procedures. These developments negatively impact physicians’ professional practices, patient safety, and overall healthcare expenditure and have emerged as “profound” issues with both economic and political ramifications in numerous countries. Reform of the medical damage liability system has triggered extensive controversy among both the medical and legal communities. Patients have raised questions regarding the rationality and effectiveness of existing policies, with the majority of debates and opposing arguments centering on “liability theory”—specifically, the efficiency and fairness of the liability framework. The impact of legislation-reforming medical damage liability on the prevention of medical malpractice must be considered; undermining claims incentives for medical improvement is highly inappropriate. Scholars have advocated integrating patient safety strategies with the regulation of medical damage liability (3), striking a balance between protecting patient rights and encouraging innovative medical practices (4). In the absence of alternative incentive mechanisms, policymakers should exercise caution when relaxing medical tort liability standards (5).

The core objectives of medical malpractice liability law include compensating patients who have sustained harm due to the negligence of medical providers, realizing corrective justice accurately, deterring improper conduct by medical providers, and curbing unsafe clinical practices. To date, national legislatures and judicial bodies have not yet established a fully feasible and internally cohesive framework of medical liability rules. It remains ambiguous whether existing medical liability systems can effectively incentivize physicians to fulfill their duty of care and curb the overuse of defensive medicine. Currently, the medical liability system remains among the most pressing challenges for the physician community, exacerbating the estrangement between physicians and patients. Physicians are constantly exposed to the looming risk of legal liability, and defensive treatment decisions prioritizing litigation avoidance over patient benefit are pervasive. Widespread anxiety regarding medical liability drives up healthcare expenditures and, to a certain extent, gives rise to accessibility and affordability barriers to medical services (6). With the continuous evolution of the global healthcare system, safeguarding patient rights and upholding the professionalism and operational efficiency of medical practitioners have emerged as critical priorities. Against this backdrop, a well-defined legal framework governing medical adverse events and professional liability has become increasingly indispensable. Varied mechanisms for allocating medical liability directly affect both patient safety and the overall quality of medical services.

Addressing global medical crises, coordinating doctor–patient relationships, and establishing standardized medical damage dispute resolution and relief mechanisms represent shared challenges for the entire world (7). Accordingly, constructing a damage liability system that conforms to social justice and clarifying the effects of liability system adjustments on damage claims, clinical decision-making, medical innovation, medical expenditures and patient safety constitute critical research topics. The issue of social justice inherent in liability allocation for medical damage is a core research theme in the interdisciplinary study of medical science and legal theory. To date, the pressure of medical malpractice liability, as measured by indicators such as medical malpractice insurance premiums, claim frequency and claim severity, as well as whether medical liability can contribute to curbing the future growth of medical expenditures, remains insufficiently explored. The social justice dimension of medical damage compensation liability is neither a purely private issue concerning routine clinical practice norms nor a simple dispute over tort liability. Instead, it is a comprehensive social issue closely intertwined with a country’s doctor–patient relationship, medical system, legal environment and other institutional factors. Medical justice is the concrete embodiment of social fairness and justice in the healthcare sector, and it should effectively mitigate power imbalances between physicians and patients to foster harmonious doctor–patient relationships (8).

Multiple considerations are needed from both policy and legal perspectives to generate novel theoretical frameworks for comparing and evaluating the fairness of medical liability systems. Only by adopting a justice-centered analytical framework can we delineate the appropriate positioning and function of laws and regulations within the public health governance system (9). Justice for medical damage compensation involves the appropriate selection and coordinated balancing of multiple values within the domain of medical damage compensation (10). The theory of justice facilitates the evaluation and critical analysis of medical accountability while advancing practical and feasible policy and institutional recommendations. The attainment of health justice constitutes a core objective for nations seeking to establish robust, equitable healthcare systems (11). The purpose of this research is to explore the imbalance between justice theory and medical damage liability, as well as the corresponding correction mechanisms. This relationship requires theoretical rethinking to resolve the dilemma caused by the long-term prioritization of individual fairness over social justice. This study aims to address pressing current issues in the medical and legal domains, propose actionable suggestions for the effective implementation of existing medical damage compensation liability regulations, alleviate doctor–patient conflicts, and facilitate the construction of harmonious doctor–patient relationships. This study investigates the constituent elements of medical damage compensation liability, causal attribution, interest balance, medical liability insurance, and doctor–patient relationships.

Drawing on specific case examinations and theoretical innovation, this research conducts cutting-edge interdisciplinary research spanning medicine, public policy and law. By comparing the different regulatory approaches adopted by China and other jurisdictions to address relevant issues, as well as their respective effects on patients and healthcare providers, this study extracts best practices for hospital accountability system construction, institutional capacity building, healthcare quality improvement and unresolved dispute resolution, which carry considerable comparative legal reference and academic theoretical value.

This study adopts the progressive logic and analytical framework of “problem anchoring---systematic---attribution---theoretical---innovation---system reconstruction.” First, it decomposes the abstract philosophical construct of the “social justice dilemma” into three observable specific dimensions: 1. the effects of excessive litigation, defensive medicine, and physician–patient confrontation on the healthcare system; 2. the issue of medical liability allocation under causal uncertainty; and 3. the challenge of reasonably distributing medical risks and realizing corrective justice. These three dimensions collectively reflect the core problems of systemic bias and structural imbalance and clarify the potential locations of justice failure in judicial rulings.

Second, this study adopts dynamic systems theory to unpack the institutional roots of justice dilemmas in the allocation of medical damage liability. Under the framework of social justice, it investigates the dynamic equilibrium among patient damage compensation, medical practice freedom and social costs and reconciles the protection of patient rights with the incentive for healthcare providers to uphold high standards of care. The objectives of the medical malpractice liability system are multifaceted, necessitating a comprehensive evaluation of the weight and intensity of each constituent element and their corresponding potential legal outcomes.

Third, accurate identification of causal relationships in medical injuries constitutes a critical step in the resolution of medical disputes. The flexible formulation of standards for proving factual causation serves as a “regulating valve” for delimiting medical liability. It is necessary to draw a clear distinction between factual causation and legal causation, and proportional causation theory has substantial theoretical value and practical significance for addressing challenges in the proof and determination of factual causation. Causation rules form an integral part of legal attribution; on the basis of established factual causation, they constitute a core principle governing liability relationships between patients and healthcare providers.

Finally, a redistribution and risk-sharing mechanism for medical tort compensation is constructed within a justice-oriented framework. It advocates moving beyond the conventional “compensation-centric” liability paradigm, shifting the adjudication logic from “outcome-based liability attribution” to “multistakeholder shared responsibility.” This approach maintains a balanced interest relationship between physicians and patients in medical damage cases, incentivizes healthcare institutions to strengthen their ethical obligations for risk prevention and control, and leverages medical liability insurance to resolve disputes and diversify social risk.

Ultimately, this paper aims to provide theoretical support for the “balanced responsibility” principle and the “doctor–patient community” concept, with the goal of fully realizing the theoretical value and practical significance of the medical damage liability system. The medical damage liability system does not exist in isolation but is closely intertwined with a variety of contextual factors specific to a given country, including doctor–patient relationships, healthcare system mechanisms, and the legal environment. Guaranteeing patient safety is a global priority, and medical liability systems around the world are currently confronting new challenges and common dilemmas related to social justice, which has triggered widespread reflection on relevant policy frameworks. While medical liability systems have distinct regional characteristics, their inherent rationality and theoretical value can provide empirical evidence and analytical frameworks for comparative law research and institutional reference, thereby deepening the understanding of the nature and operational rules of medical liability.

## Justice logic and practical challenges in medical liability allocation

Medical damage liability disputes constitute the primary manifestation of doctor–patient conflicts. They not only directly impact the protection of patients’ rights to life and health but also profoundly influence the high-quality development of the medical industry as well as social harmony and stability (12). With increasing awareness of citizens’ rights protection, the number of medical damage liability disputes has been growing annually, and this has become a key bottleneck restricting the healthy development of the healthcare industry (13). The Medical Malpractice Act is designed to allocate liability to healthcare providers who fail to comply with established clinical care standards and thereby cause patient injury, with the core objectives of compensating for injured patients, penalizing negligent medical practitioners, and deterring medical negligence. However, the current liability system has failed to achieve its intended effects; conversely, it impedes initiatives to improve patient safety and may even increase the incidence of adverse medical events. Empirical evidence and widely publicized, socially alarming cases of medical harm demonstrate from multiple dimensions that existing doctor–patient dispute resolution mechanisms have undermined the realization of social justice. The modern conception of social justice constitutes a systematic framework of fairness centered on distributive justice (14, 15). Against this backdrop, medical liability system reform has attracted extensive attention from academics and the public. Clinical, technical, legal and policy-oriented challenges have created clearer directions and broader scopes for advancing social justice in the medical field.

### The impacts of excessive litigation, defensive medicine, and doctor–patient confrontation on healthcare systems

Medical disputes represent a global public health challenge. The inherent uncertainty and risk-based characteristics of clinical practice may induce a vicious cycle of overlitigation and defensive medicine (16). With the increasing application of invasive medical technologies in clinical practice, the safety risks of medical services have correspondingly increased, leading to a substantial incidence of adverse medical events (17). In the context of frequent medical damage disputes and the growing volume of medical damage compensation cases, the development of China’s healthcare sector as well as social harmony and stability face threats (18). Amid the rapid expansion of medical and health services, medical incidents are occurring with increasing frequency, the financial burden of medical injury compensation has become unsustainable, and doctor–patient conflicts continue to intensify (19). Patients have sustained preventable injuries that have fundamentally altered both their lives and their trust in healthcare systems. Owing to the inherent uncertainty, potential hazards, and intrinsic complexity of medical discipline, discrepancies frequently emerge between patient expectations and clinical practice (20). Existing related research, which analyzes case volume, spatial distribution, case typology, court hierarchy and judgment document categories, concludes that medical damage liability disputes are characterized by large case volume, rapid growth in case numbers and high case complexity (21). The identification of medical professional issues in medical disputes does not fully comply with the principles of peer review and lacks closed-loop management between dispute resolution and prevention from the perspective of “patient safety” (22). It is imperative to standardize doctor–patient relationships and balance the rights and obligations of both parties. While substantial empirical evidence confirms the role of medical malpractice liability systems, identifying the specific effects of such systems on physicians’ clinical decision-making remains a challenging research task.

In the United States, healthcare providers face approximately 85,000 medical malpractice lawsuits annually, and medical malpractice is estimated to cause more than 250,000 deaths each year, ranking as the third leading cause of death in the country, following heart disease and cancer (23). The medical malpractice crisis has given rise to challenges in terms of the affordability of medical malpractice insurance, thereby limiting patients’ access to healthcare services (24). Medical liability insurers are exposed to substantial liability risks stemming from medical malpractice claims, with the annual aggregate cost of the U. S. medical liability system approaching $56 billion (25). Litigation and violence against medical institutions erode the quality of healthcare delivery, compromise the regular provision of medical services, and inflict substantial economic losses on the healthcare sector. The indirect costs generated by the current medical liability system far outpace those incurred by medical malpractice compensation, and the expansion of medical malpractice liability drives a notable increase in overall medical expenditure. When medical institutions lose medical malpractice litigation, in addition to compensating for damage directly attributable to the institution’s liability, nearly 79.3% of adjudicated cases also award compensation for noneconomic damage to the patient and their family members (26). Medical disputes are directly or indirectly negatively correlated with occupational efficacy among healthcare workers. This issue severely impairs healthcare workers’ physical and mental health, triggering negative outcomes, including anxiety, depression, and occupational burnout, and reducing job satisfaction. It also increases the incidence of defensive medical practices among physicians and ultimately compromises the overall quality of medical services. According to a 2018 survey released by the Chinese Medical Doctor Association, 62% of practicing physicians have encountered medical disputes of varying severity, and 70.67% of respondents attribute their high levels of work pressure to three main sources: medical disputes, heavy workloads, and excessively high patient expectations (27). The increasing incidence of medical malpractice allegations has elicited substantial concern within the medical community, as stress disorders secondary to medical malpractice incidents have rendered physicians indirect victims of such events (28). A 2021 physician survey published by Tsinghua University indicated that 57.95% of respondents reported being fearful or extremely fearful of becoming the target of medical disputes, and as many as 31.0% explicitly stated that they would avoid accepting high-risk patients to prevent doctor–patient conflicts (29). The strained doctor–patient relationship severely undermines the legitimate rights and interests of both parties and impedes the sound development of the healthcare industry.

Medical practice is characterized by its exploratory nature, empirical nature, specialized technicality, risk uncertainty, and the inevitability of adverse harm. Increasing the burden of proof and corresponding legal responsibility on medical providers can induce issues such as the abuse of litigation rights by patients, defensive medicine, and excessive medical examinations. Empirical behavioral data indicate that fault liability pressure does prompt physicians to opt for more medical services; regardless of the specific payment system in place, this pressure has intensified to the extent that it has given rise to established “defensive medicine” practices (30). Defensive medicine is spreading globally and may trigger risks that disrupt healthcare systems. The generalization of damage liability for medical malpractice tends to induce the emergence of defensive medical practices (31); furthermore, “excessive medical treatment is essentially a specific and relatively independent medical tort” (32, 33). In April 2002, the Several Provisions of the Supreme People’s Court on Evidence in Civil Procedures were officially promulgated. Article 4, Item 8 of the Provisions clearly stipulates the reversal of the burden of proof for medical tort cases, which reduces the litigation costs and the burden of proof for patients. Nevertheless, under the resulting pressure, some medical institutions have entered a stage of “defensive medicine” practice, which has given rise to overtreatment and inappropriate refusal of patients. Numerous physicians report that they face psychological pressure stemming from the fear of bearing liability, and this fear consequently induces excessive prescription, medical testing and overtreatment (24).

The medical liability system is commonly recognized as a core contributor to rising healthcare expenditures, as it incentivizes the practice of defensive medicine. In this context, physicians intentionally overutilize low-cost-effectiveness healthcare services to mitigate their exposure to financial liability and fault-based liability risks. Specifically, defensive medicine refers to preventive medical interventions adopted by clinicians to avoid medical risks and litigation, motivated by considerations of self-interest. In this scenario, clinical decision-making is primarily or even entirely driven by the fear of medical malpractice claims rather than being guided solely by the patient’s individual clinical condition. This practice prioritizes the minimization of litigation risk over the protection of patients’ interests and rights (34). Patients are frequently subjected to unnecessary examinations and associated potential harms, as clinical decision-making fails to prioritize patients’ best interests (28). This practice frequently exposes patients to unnecessary examinations and potential hazards but fails to take into account the best interests of patients (28). This defensive behavior exerts pressure on the entire healthcare system: it continuously drives up healthcare costs, creates a backlog of pending cases, restricts patients’ access to medical services against established expectations, and limits access to treatment for individuals with legitimate medical needs. Previous studies have reported that the widespread use of defensive medicine is responsible for the global escalation of health care costs (35). Extensive research has identified defensive medical motivation as a core driver of rising healthcare costs (36). Although specific quantitative figures are difficult to determine, defensive medicine may contribute approximately 2.8% of total healthcare expenditures (37). Empirical evidence indicates that the practice of defensive medicine fails to generate quantifiable health benefits and has negative effects on clinical disease management and the protection of patients’ rights and interests.

The inherent attributes of the healthcare industry confirm that legal liability has a substantial influence on physicians’ decision-making mindsets and clinical practices. Specifically, the regional legal regulatory environment shapes both physicians’ clinical behaviors and patients’ access to medical care services. Avoidance of medical litigation is recognized as the direct incentive for the emergence of defensive medicine, and this phenomenon is commonly attributed to the current medical tort liability system (6). Apart from medical malpractice litigation itself, few issues in healthcare governance elicit more intense controversy than defensive medicine does. To date, the adverse externalities of defensive medicine have emerged as a shared challenge confronted by global healthcare systems and constitute the core rationale for reforming existing medical malpractice liability regimes.

### The issue of allocating medical liability under factual causation uncertainty

Medical liability stemming from causal uncertainty precludes clinicians from fully elucidating and controlling the rationale underpinning their clinical decisions and the codes of conduct that govern routine clinical practice. This uncertainty gives rise to fundamental concerns regarding procedural fairness: the “diffusion of responsibility” not only undermines legal certainty but also incentivizes medical practitioners to evade accountability for critical clinical decisions. In such contexts, the deficiency of judicial fairness originates from the ambiguity surrounding the attribution of liability and the identification of accountable parties, which ultimately induces substantial deviations from established overall care standards and exposes long-standing challenges regarding the demarcation of medical liability boundaries and the realization of fairness within healthcare systems.

One of the core objectives of the medical liability system is to improve the quality of medical services by deterring negligent conduct. To achieve this goal, the tort law system must maintain accuracy in both fault identification and causation determination. However, existing empirical data reveal substantial discrepancies in causal assessment between actual medical malpractice incidents and patient harm: approximately 80% of medical malpractice claims lack probative evidence to establish causation (38). In most judicial adjudications, where “causal contribution” (the degree of contribution to the harmful outcome in medical malpractice) is identified, the medical provider is ruled to bear corresponding tort liability. Improper application of “causal contribution” will adversely affect the ascertainment of legal causality. In essence, the proportion of liability corresponds to the scope of liability, and the determination of factual causation required for the establishment of liability must be completed before the division of the proportion of liability (38). Attempting to negate the factual causation of tort liability by referencing the “degree of participation” or “causative potency” provided in forensic appraisal opinions introduces practical difficulties and conceptual ambiguities to probability-based factual proof. Currently, many courts’ judicial review of appraisal opinions remains merely procedural: courts often directly copy appraisal conclusions in judicial rulings without substantive verification, and the ambiguity of the civil proof standard inevitably leads to the inappropriate application of the rule that “liability follows fault.”

In China, the plaintiff victory rate in medical tort litigation is 79.5%, yet plaintiffs seldom obtain full compensation for their damage; among all victorious plaintiffs, 94.8% receive only partial compensation (39), resulting in ambiguity regarding the application of causation standards (40). In contrast to the United States, where only 25% of plaintiffs prevail in medical tort litigation (41), only a very small proportion of adverse events caused by medical negligence ultimately lead to medical malpractice claims (24), and lower expected compensation also undermines the deterrent effectiveness of the medical malpractice liability system. Taking the judgments on medical damage liability disputes concluded between 2020 and 2022 by Jiangxi Provincial Courts and published on the China Judgments Online website as the research sample, this paper analyzes the courts’ final determination of participation in medical errors (causative potency). The results show that only approximately 3% of medical technical damage liability cases are ruled out as having no causal relationship, while 97% of cases require medical institutions to bear liability for medical errors, which demonstrates that a high proportion of medical institutions are liable for compensation (42). Numerous studies have demonstrated that causal inference strongly depends on the completeness of the evidence chain, and this represents a pervasive challenge in contemporary medical malpractice liability disputes (43).

Medical practice involves substantial inherent uncertainty, particularly since numerous diagnostic and therapeutic interventions themselves carry iatrogenic risks. These findings indicate that any therapeutic intervention administered by medical personnel has a nonnegligible risk of inducing bodily harm to patients. If the threshold for qualitative change in causal attribution is overlooked, medical personnel are at heightened risk of being held erroneously liable. This outcome may erode their courage and decisiveness in managing intractable diseases or prompt defensive practices and overtreatment as self-protective measures. Medical injury cases involve extremely high professional specificity: whether clinical diagnosis and treatment constitute malpractice, whether a causal association exists between clinical practice and patient harm outcomes, and the magnitude of causative potency typically require formal medicolegal expert appraisal (44). Although certain appraisers at appraisal institutions hold a medical education background consistent with that of practicing clinicians, they frequently lack adequate clinical practice experience. Moreover, the appraisal process adopts reverse reasoning—that is, it conducts retrospective analysis and judgment on the basis of existing outcomes. Consequently, the validity of its conclusions regarding the risk level and clinical significance of various diagnostic and therapeutic behaviors, the causal association between these behaviors and clinical outcomes, and the scientific rigor of the underlying identification models remain questionable (45). The rule of allocation for medical damage liability, which has long been established and applied in China’s judicial practice, enables medical institutions to apportion losses with patients. Current rules governing causal evidence undermine the ability to predict the systematic identification of medical malpractice, and recognizing the significance of addressing this issue is necessary.

### Rational allocation of medical risks and the dilemma of realizing corrective justice

Medicine is an ever-evolving discipline. Only through sustained innovation and exploration in disease diagnosis and treatment can clinical standards be elevated. Medical risk prevention primarily relies on healthcare practitioners’ vigilance toward medical risks and the implementation of targeted preventive measures. Medical adverse events are not solely induced by medical negligence; they also include nonnegligent events such as complications, progression of underlying diseases, interindividual patient variability, and unpredictable adverse outcomes. Not all the harm experienced by patients falls within the scope of compensation for medical malpractice, and indiscriminately imposing liability on medical institutions not only impedes routine clinical practice but also leads to the unlimited expansion of the scope of institutional liability (46). Owing to the complexity of the etiology of medical damage, patients incur substantial time costs when they undergo medical appraisal and initiate litigation. After medical damage is sustained, patients often face sharp confrontations with medical institutions. Furthermore, the approach of concentrating medical risk-bearing on a single entity impedes hospitals from proactively engaging in the innovation of medical technologies, which is detrimental to medical advancement. To reduce medical expenses, hospitals frequently opt for conservative treatment schemes, which leads to the risk of transferring patients with various difficult and severe conditions to external medical institutions (Error! Bookmark not defined.). Given the limitations of current medical technology, when faced with complex and variable diseases, existing medical risks already exceed the capacity of individual undertakings. Neither hospitals nor patients can bear such substantial medical risks alone. The public welfare attribute of medical practices determines that their legal value assessment should be prioritized from the perspective of the overall interests of society. As a globally recognized sector characterized by high risk, high technical difficulty and heavy professional responsibility, healthcare is confronted with doctor–patient conflicts and even medical crises, which are pressing social issues facing all countries around the world.

The pooling of medical risks by social forces embodies the value of social equity. This mechanism not only helps reduce the social cost of bearing medical risks but also facilitates the proper resolution of doctor–patient disputes (47). Existing research has demonstrated that the implementation of compulsory medical liability insurance, as a market-oriented financial innovation mechanism, enhances the completeness of the medical service market, effectively promotes trust and cooperative relationships between physicians and patients, and significantly reduces the incidence of medical disputes (19). Liability insurance can mitigate defensive medical practices, reduce healthcare expenditures, and strengthen trust between physicians and patients, representing an effective Pareto-improving institutional arrangement for multiple stakeholders. The emergence and development of medical liability insurance have advanced the realization of corrective justice for medical torts: it facilitates compensation access for victims and alleviates conflicts stemming from economic interests between physicians and patients. Regardless of how liability is allocated, insurers typically bear and pool associated costs. However, it should be noted that in noncompetitive healthcare markets where patient demand is inelastic, liability costs are ultimately passed through to patients. Healthcare providers may shift the premium costs of medical malpractice liability insurance to patients via higher service prices, with patients being left to bear the primary cost of risk mitigation (48). This fully socialized damage compensation system facilitates the realization of the general preventive function of tort liability law.

Patient safety constitutes the core objective and fundamental mission of the entire medical system and should serve as the fundamental purpose of medical liability insurance. Assuring patient safety in health care is a critical component in delivering the right to health. 1 in every 10 patients experience harm in healthcare; approximately 50% of this harm is preventable” (1). Patient safety serves as an indicator of the degree to which a state upholds, protects, and fulfills its overarching commitments to health-related human rights (1). Within the healthcare system, the doctor–patient relationship is shaped by the normative framework governing the rights and obligations of both parties (28). The medical damage liability system is widely recognized to fulfill three core functions: compensating victims of medical malpractice, delivering corrective justice to affected parties, and incentivizing high-quality clinical care via deterrence effects. When evaluating medical liability legislation, it is necessary to adopt a perspective centered on unreasonable risk prevention, assessing two key dimensions: whether the existing framework is insufficient to deter physician misconduct and whether it may inadvertently increase the incidence of medical malpractice. By adjusting and optimizing physician–patient relationship governance and establishing an efficient accountability mechanism for processing claims arising from medical malpractice, patients can obtain fair and appropriate compensation without rendering the treating physician an unintended collateral victim of the system (28). However, assessing the extent to which medical tort liability law attains its intended policy and normative objectives remains a methodologically and conceptually challenging inquiry.

The core mission of tort law is to reconcile the tension between the protection of legal interests and freedom of conduct, given that both of these values are constitutionally protected fundamental rights. Tort law constitutes not only a remedial regime for rights and interests but also a normative framework that safeguards freedom of conduct (49). The objectives and values of the medical tort liability system are multifaceted: It not only protects the rights and interests of patients but also preserves and promotes the healthy development of medical services and prevents excessive liability risks from being imposed on physicians. A more patient-biased liability system inevitably leads to a surge in the number of claims, which in turn gives rise to defensive medicine issues associated with medical liability. Overprotection of patients further restricts or suppresses the freedom of medical practice, breaking the original balance of interests between the two parties. Regardless of how the “fair sharing of losses” is defined, such overprotection essentially imposes undue constraints on the freedom of medical practice. Since compensation for medical damage is established as a form of tort liability, it should follow the principle of proper coordination between professional freedom and public safety under tort law. As a constraint mechanism, law fundamentally aims to prevent excessive interference with individual freedom. Liability norms must intervene in the freedom of medical practice in proportion to the magnitude of risk rather than deviating from the basic principles and institutional positioning of tort liability. Such deviation will lead to indiscriminate judicial remedies for patient damage, resulting in the neglect of protection for medical freedom and new forms of unfairness.

It is imperative to ensure the balanced design of patient rights protection channels and promote the equitable allocation of responsibilities among multiple stakeholders to sustain fairness and justice in the healthcare domain (50). This approach safeguards fairness and justice in the medical sector and enables the broader social justice framework to incorporate a sound conceptualization of medical justice (51). It is necessary to construct a “multifaceted framework for health justice” grounded in five value dimensions: equality, liberty, individual respect, utilitarianism and responsibility (11). Liability for medical damage compensation differs from general tort liability for damage, as it pertains to the maintenance of national medical order and constitutes a core component of the deepening of social governance reform. It necessitates the balancing of interests among individual patients, the medical industry, and the overall public interest.

## Dynamic balance among patient damage compensation, medical freedom and social costs under the concept of social justice

The health sector constitutes a fundamental issue requiring shared responsibility across social, political, and legal domains. The concept of “public health” encompasses a broad spectrum of distinct constructs, professional identities, health policies, institutional responsibilities, scientific methodologies, and ideological perspectives. Public health legal scholarship should assume a more prominent role in this domain, specifically through active integration with the philosophy of social justice and the alignment of justice conceptions, legal assessment frameworks, and medical liability legislation (9). Law is a system of normative rules, and legal scholarship itself furnishes a crucial framework for the critical evaluation of justice in accordance with value standards (9). The intervention of public interest factors alters the interest configuration between physicians and patients, substantially reshaping the inherent logic of risk allocation. Furthermore, the evolution of legislative norms inherently reflects the value orientation of medical tort liability systems, which has gradually shifted from the unipolar protection of “patient rights” toward the dynamic balance of “patient compensation–medical autonomy–social cost” (52). The concept of a “community of shared destiny between doctors and patients” constitutes an in-depth reflection on the current strained doctor–patient relationship and represents a redefinition of the development pathway for constructing harmonious doctor–patient relationships (53). In the early developmental stage, greater emphasis was likely placed on patient rights. However, against the backdrop of the social welfare attributes of the medical industry, the contemporary value orientation of healthcare governance is transitioning from an individual-centered remedy to the pursuit of overall social efficiency. Accordingly, the traditional one-dimensional framework of patient protection requires corresponding adjustment, and medical institutions and practitioners should legitimize their clinical practice through the fulfillment of social responsibilities. The existing medical liability regime necessitates fundamental adjustment and structural reform. The monistic framework of health justice has inherent critical flaws; thus, the exploration of new justice criteria for healthcare systems from multidimensional perspectives is needed. Substantial uncertainties, contradictions and tensions exist between tort law remedies for rights and freedoms, between high-quality healthcare accessibility and economic affordability, and between the normative logic of tort law and overarching social objectives, rendering this issue a complex exercise of legal and policy coordination. Balancing the tripartite interests of physicians’ practice autonomy, patient safety and the economic affordability of medical services is inherently complex yet indispensable and ultimately contributes to a dynamic equilibrium embedded in the “spatiotemporal” conceptual framework.

### Strike a balance between protecting patients’ rights and incentivizing healthcare providers to sustain high standards of care

Globally, suboptimal decision-making in clinical practice has emerged as a core contributor to medical disputes. An analysis of liability attribution in adjudicated medical decision-making litigation indicates that medical institutions bear secondary liability in 52.3% of clinically adjudicated fault cases, while primary or full liability accounts for only 25.1% of the total. This distribution reflects the inherent particularity, complexity and unpredictability of diseases, as well as the inherent uncertainty of clinical interventions in the medical field (54). Patient safety is defined as the prevention of preventable harm to patients throughout the delivery of healthcare services, encompassing the reduction of risk associated with avoidable medical harm to an acceptable threshold (1). The intersection of patient safety and legal accountability has never been of greater significance, as healthcare quality and safety have emerged as core indicators of national healthcare system development. Hospitals deliver safe, regulation-compliant patient care that consistently operates at the dynamic interface of clinical practice, statutory requirements, and evolving judicial frameworks.

In recent years, global attention to medical and patient safety has risen steadily, driven predominantly by the persistent burden of preventable harm and medical errors, alongside the demand for robust judicial systems and supportive legal infrastructure. Nonmaleficence, justice, and respect for patient autonomy constitute the foundational ethical principles of medical practice. While advances in medical technology and managerial innovation are required to improve treatment success rates, excessive deterrence incentivizes defensive medicine and constrains clinical autonomy and innovation, creating an imperative to reconcile these competing value objectives. Accordingly, integrating medical quality, patient safety, and healthcare management with the governance of medical harm and malpractice claims has substantial scholarly and practical value. This integration, however, must be premised on comprehensive protections to prevent unfair reputational harm to practicing physicians (55). The intricate landscape of legal liability imposes sustained impacts on clinical practice, necessitating a delicate balance between the professional obligations of healthcare practitioners and patients’ rights.

Advancements in medical science have continuously expanded the scope of available treatment options. However, this expansion is increasingly associated with substantial growth in the costs of medical equipment and pharmaceutical products. Emerging therapeutic paradigms often present a dilemma between medical progress and economic affordability, requiring that the probability of successful treatment outcomes be weighed against the potential risks and burdens that the intervention imposes on patients (56). In response to cost control mandates, physicians and healthcare institutions actively deliver cost-conscious medical services, aiming to reconcile the competing legal and economic societal expectations regarding healthcare provision and expenditure management. The overarching aim of UHC is for all people who need health services to receive high-quality care without financial hardship. Quality health services (promotive, preventive, curative, rehabilitative and palliative) are thus embedded within the definition of UHC (57). The medical liability system generates heterogeneous pressure on healthcare providers, which provides exceptionally valuable empirical data for investigating the actual operational mechanisms of tort law systems. Furthermore, medical liability does not necessarily induce a socially optimal level of precaution, as incentive mechanisms are distorted through multiple pathways (58). If the pressure imposed by fault liability induces excessive precaution, such a degree of precaution exceeds the legally required standard of care and fails to achieve the socially optimal level of care. Medical malpractice liability enables patients to file claims and secure compensation and may incentivize negligent physicians to adjust their clinical practices and upgrade treatment standards. However, defensive medicine driven by the pressure of medical liability may offset the corrective effect of liability rules on aggressive practices.

A fundamental tension exists between the freedom of medical practice and patient medical safety, and the core mandate of law is to achieve interest balance. There is an inherent need to reconcile the incentive effect of liability regimes with patient safety and medical innovation. This process involves multiple heterogeneous stakeholders, rendering the balancing exercise inherently complex and sustained. The healthcare system cannot be fully transformed into an industry driven solely by economic incentives: it constitutes a critical and indispensable public service that requires rigorous regulation, and patient safety must be prioritized over economic interests. To guarantee the safe provision of medical services and medical products, the establishment and effective enforcement of sound legal and regulatory frameworks is essential for all countries (59). Regardless of the type of medical damage liability regime adopted, the common prerequisite is that medical institutions and practitioners pursue their interests in a reasonable and restrained manner. The wide-ranging impact of the commercialization of physicians and the medical industry is reflected mainly in the weakening of self-regulatory norms within the medical profession and the corresponding increase in medical negligence incidents (60). If profit replaces medical services as the primary objective of hospitals and physicians, the normative character of medical practice will inevitably be constrained. This creates conditions for economic motives to be concealed behind the cloak of professional independence; hence, the economic incentives within the medical community should be minimized.

If critical mutual trust between physicians and patients is compromised, it inevitably undermines physicians’ work motivation. Consequently, physicians become risk averse toward clinical decision-making: they tend to overlook potential symptoms and avoid high-risk treatment options as much as possible. Therefore, it is necessary to emphasize the importance of safeguarding the legitimate rights and interests of physicians (61). Dynamic balancing mechanisms between fault deterrence and free innovation, as well as between medical cost control and medical liability under spatiotemporal constraints, represent the development trend of medical malpractice legislation. To address the legal dilemma of the fiduciary relationship between physicians and patients, the principle of “interest equity” should be upheld. Specifically, strengthening patients’ right to benefit and right to supervision can offset their legal status and factual evidence disadvantages, thereby reducing the possibility that medical institutions and practitioners abuse fiduciary rights and discretionary powers (62). Defensive medicine encompasses not only the provision of unnecessary examinations and treatments but also the withholding of treatments deemed inconsistent with professional practice by judicial authorities (63). If clinicians can confirm that patients have provided explicit informed consent to receive innovative treatments and that clinicians are exempted from liability for appropriate implementation of such treatments, many urgently needed medical innovations can be deployed more rapidly rather than being stalled, ultimately delivering overall benefits to patients (64). A core constraint encountered by physicians is the restriction imposed on their autonomy in clinical decision-making: physicians retain a degree of discretion when multiple equivalent, clinically justified and innovative intervention approaches are selected and are not required to uniformly opt for the safest treatment option. Given the primacy of patient welfare and the documented drawbacks of conventional standardized treatment, the adoption of innovative clinical methods is ethically and clinically justified (64).

Research on the medical responsibility system has shifted from postincident remediation to prevent risk prevention and control, with its positioning centered on safeguarding patients’ lives and health. This shift enables diagnostic and nursing approaches to effectively address the principal contradictions of a patient’s condition at the clinical stage. While medical decision-making by physicians may involve erroneous judgments that lead to adverse outcomes, it is necessary to avoid irreversible harm to patients caused by subjective negligence on the one hand and, on the other hand, not to impede legitimate clinical innovation and evidence-based risky attempts by physicians. Furthermore, physician overconfidence can have positive effects, as it helps foster motivation, sustain resilience in treatment, and support the willingness to undertake reasonable clinical risks. Ultimately, preventing and reducing adverse medical events requires medical teams to enhance clinical technical capabilities and improve institutional risk-sharing mechanisms (65). An indispensable balance must be struck between efficiency and safety, as well as between innovation and responsibility, to establish a medical accountability system that accommodates advances in medical technology without compromising patient dignity and safety (66). Therefore, a reasonable boundary must be established between cultivating adequate awareness of medical error prevention among clinicians and avoiding the impediment of promising, groundbreaking medical innovations. The obligations and liabilities of medical providers should be defined and expanded in a reasonable manner: this framework should neither sacrifice the legitimate rights and interests of patients by denying them access to appropriate medical care nor restrict the innovative development of medical institutions and their personnel. Maintaining this reasonable boundary is a core component of safeguarding patient safety and increasing clinical cure rates.

### Clarifying the institutional dilemma and boundaries of medical damage liability under the framework of the “community of shared interest”

Unlike ordinary civil disputes, where the two parties typically exhibit a “zero-sum game” dynamic, medical disputes distinctly demonstrate the nature of a “negative-sum game.” Medical practices feature a certain degree of positive externality, with the core objective of eliminating or alleviating human illnesses and improving public health. If medical injury occurs during treatment and escalates into a dispute, it not only inflicts health harm on patients but also may even lead to patient death, resulting in varying degrees of economic loss and psychological trauma to both patients and their families. The vast majority of medical personnel hold regret and sympathy for such outcomes; even in cases where they bear no fault, many experience genuine guilt over the objective occurrence of the injury. The occurrence of medical injury also constitutes a heavy blow to medical personnel. Therefore, extracting prevention experience from medical disputes to avoid the recurrence of such risks in future practice is the only positive outcome that can be derived from medical disputes. The doctor–patient relationship is an inevitable fundamental factor in the construction of medical damage compensation liability. The core of properly resolving medical disputes lies in fundamentally clarifying and understanding the nature of the doctor–patient relationship.

The doctor–patient relationship is a “community of shared interests” where both parties share gains and losses, an interest bond tied to the common goal of “combating illness and accelerating patient recovery.” It is a social interest structure that incorporates private interests, an ethical relationship rooted in objective interest connections, and a social relation with public interest attributes. One of the core root causes of medical disputes lies in the changing perception of the nature of the doctor–patient relationship (62). The blurring of doctor–patient boundaries can have negative effects on both physicians and patients. At present, the construction of a community with a shared doctor–patient destiny still faces multiple legal challenges, including imbalanced allocation of rights and obligations, insufficient protection of doctor–patient rights and interests, and imperfect medical dispute resolution mechanisms (54).

Medical practitioners and patients share the same core goal of disease treatment, which is a defining premise for the medical liability system and directly influences the allocation of “responsibility” under medical damage compensation liability. Unlike the general tort liability system that regulates private interest relationships between individual private parties, the medical damage compensation liability system determines that the resolution of medical damage disputes must incorporate the legal interest objective of protecting public social interests. In the doctor–patient relationship, patients entrust their health and even the right to life to medical practitioners. For such fundamental rights, physicians must assume both legal obligations and ethical responsibilities. Health and life are of irreplaceable value to all human beings; therefore, the obligations and rights between doctors and patients cannot be interpreted as an equivalent compensable exchange of private interests. Otherwise, the doctor–patient relationship would degenerate into a pure transaction of rights in exchange for monetary gain. Patients transfer control over their bodies and health rights to medical institutions and practitioners to leverage the latter’s professional competence to pursue benefits for patients. Medical practice must comply with the fundamental principle of “maximizing patients’ interests,” and medical staff should not seek illegitimate interests for themselves or third parties beyond collecting reasonable medical expenses (63). This inherent characteristic of the doctor–patient relationship determines the likelihood of medical damage. The expectation that assigning liability burden to medical providers can effectively reduce patient harm is largely ineffective, and such damage requires comprehensive remedies for patients’ rights and interests to achieve adequate redress.

Medical harm is an unavoidable outcome inherent to the clinical process. Its occurrence is independent of variations in medical technology, resource endowment, institutional management, or the subjective and objective intentions of both clinicians and patients. As a consequence of the interaction of multiple confounding factors, it is often challenging to clearly attribute legal liability to faults. The consequences of medical harm are severe and multifaceted: medical harm also inflicts substantial damage on clinicians, and extensive empirical evidence confirms that many clinicians have been pushed to the brink of career termination because of medical litigation and associated compensation liabilities. Furthermore, medical harm constitutes a net social loss. Once it occurs, it transcends the bilateral scope of the clinician–patient relationship, threatens the stable operation of the healthcare system and overall social order, and exacerbates social conflicts. Restricting the allocation of liability for harm to the balance of private interests will prompt patients or medical institutions to focus only on safeguarding their own private interests. The value foundation of medical harm relief has undergone a fundamental shift, transitioning from taking private law liability as the core framework to attaching equal importance to both public law and private law liability, which constitutes the new normative basis for such relief (10). Therefore, although medical damage liability and general tort liability share isomorphic complementary relationships and overlapping attributes, they are grounded in distinct regulatory logics and liability frameworks, respectively. Their modes of liability assumption and demands for substantive justice diverge, and neither can exist independently of the current operational context of the medical system. Furthermore, constrained by the inherent attributes of medical services, the functional positioning of medical damage liability should prioritize an incentive role rather than a compensatory role, making the implicit incentive effect of relevant liability allocation particularly prominent (49).

The contemporary Chinese healthcare system integrates public welfare attributes and operational efficiency. However, driven by multiple factors, such as uneven resource allocation, regional development disparities, and heterogeneous service quality among medical institutions, the marketization of medical services and the scarcity of high-quality medical resources have eroded the public welfare of medical services to a certain extent. Given this background, after they face high medical costs, patients hold higher expectations for procedural fairness in medical services and the certainty of treatment outcomes. When liability for medical damage is defined, legislatures tend to tilt toward generalizing the protection of patients’ rights while weakening the rights protection of physicians. This imbalance in the rights and obligations of doctors and patients further triggers conflicts between patients’ pursuit of individual justice and the demand for overall social justice within the medical system. The institutional arrangement of medical damage compensation liability should transform its core focus from “liability establishment” to “right remedy,” and the right remedy should serve as the logical foundation for legislation on medical damage liability. The core normative purpose of medical damage liability is to regulate clinical diagnosis and treatment behaviors. In this context, the demand for “ensuring physicians can continuously provide high-quality medical care” and “preventing similar adverse damage events” is far stronger than the demand for monetary compensation. Traditional tort theory is constrained by the “damage filling principle” and cannot provide a comprehensive prevention and control mechanism to reconcile doctor–patient relationships and mitigate mutual trust risks (63). In the identification of medical damage compensation liability, attention should be given to the rights-related factors in medical damage disputes. Relying solely on the “restitution in kind” remedy framework provided by civil law not only fails to alleviate doctor–patient conflicts and reduce the incidence of disputes but may even trigger systemic risks to the medical service system.

Given the inherent coexistence nature of doctor–patient relationships, the allocation of medical damage compensation liability should transition from the technical constraint of “fault identification” to a substantive framework centered on “equitable treatment of both doctors and patients” to respond to patients’ demands for the protection of their rights and personal dignity. In medical dispute resolution, the plaintiff, as the relatively vulnerable party in the relationship, should receive greater consideration and institutional protection (67). On the basis of safeguarding the fundamental rights of patients, reasonable financial compensation should be guaranteed. Legislative and judicial authorities should consider increasing the amount of compensation for mental distress to align with social development (68). Patients and medical professionals are collaborative partners; high-quality clinical decisions rely not only on evidence-based scientific foundations but also on the degree of alignment between the intervention plan and patients’ individual life values (68). The publication of the Expert Consensus on Clinical Practice of Doctor–Patient Shared Decision-Making (2026) signals the transition of the clinical decision-making model from a physician-led paradigm to a patient-centered shared decision-making paradigm, which contributes to rebuilding doctor–patient trust and mitigating medical conflicts (69). The contemporary doctor–patient relationship is deeply mired in a crisis of meaning dominated by technological rationality, and there is an urgent need to transcend the existing research limitations of static structural analysis and one-way skill optimization (70) and to deliver academic interpretations of the logic, value implications and practical approaches underlying the norms of medical responsibility. Building a community with a shared future between doctors and patients is an integral component of the high-quality development of China’s medical and health sector (71).

### Dynamic systems theory of medical malpractice liability: element sequencing and intercorrelation

The medical industry is characterized by high professionalism, strong social initiative, and inherent high risk. Medical liability intersects not only with medicine, public policy, and law but also with justice-related considerations and is traditionally fault-based, arising exclusively from negligent acts that violate established medical professional norms and standards. China’s evidentiary rules for medical dispute resolution have undergone three iterative adjustments: evolving from the dual inversion of the burden of proof for both causality and fault elements to the restoration of a standard forward-looking burden of proof for causality. This evolutionary trajectory reflects the gradual deepening of legislators’ understanding of the imbalance in interest between physicians and patients. The inverted burden of proof rule once the protection of patients’ interests was excessively prioritized not only induced the unintended consequences of defensive medicine but also failed to reasonably allocate evidentiary risks arising from the inherent limitations of medical knowledge. Given the exploratory nature of medical science, the risks inherent in diagnosis and treatment should not be unilaterally assumed by a single party, and it is unreasonable to favor only the interests of injured parties. Causality in medical disputes inherently involves scientific uncertainty and does not depend on whether one party can exercise complete control over relevant evidence. This type of risk allocation should no longer disproportionately favor one party; instead, it should achieve a balance of interests between the disputing parties and incorporate evaluation of the public interest value of the medical practitioner’s conduct.

In the field of medical damage compensation, the pursuit of diversified values implies that when adjudicating discretionary liability, judges are guided and constrained by multiple values, including safeguarding patients’ rights and interests, advancing medical and health services, and fostering harmonious doctor–patient relationships (72). The exercise of judicial discretion often requires the balancing of political considerations, public opinion, the interests of vulnerable groups and social impacts, which may lead to the neglect of legal logic as well as fairness and justice (73). Behind the allocation of liability for medical damage often lies a complex balance of interests and value judgments. Dynamic systems theory emphasizes the sequential relationships and interconnections between constituent elements: The satisfaction of weightier elements can offset the deficiency of less significant elements, and the higher the factor intensity is, the greater the likelihood of generating corresponding legal effects. When dynamic systems theory is applied, it is necessary to first determine which legal principles should be adopted for element selection and then clarify the hierarchical order among different elements (73) to identify the specific determinants that influence the mitigation or exemption of liability for medical institutions, along with the magnitude of such determinants’ effects. Although dynamic systems theory remains open to multiple unresolved questions, it still serves as an effective framework for balancing pluralistic interests and coordinating heterogeneous value relationships.

By rationally defining the hierarchical order and interconnections of constituent elements, this approach enables the equitable allocation of responsibilities between physicians and patients, thereby facilitating the construction of harmonious physician–patient relationships. Recently, the American Law Institute has adopted the Restatement of the Law, Third, Torts: Liability for Physical and Emotional Harm: Medical Malpractice, which reorients the standard for determining medical negligence from the long-standing “customary industry practice” to a more substantively meaningful “patient-centered reasonable medical standard.” This review integrates evidence-based medicine and principles of evidence law into the adjudication of medical torts, establishing an updated, standardized criterion for identifying medical malpractice (74). The core objective of this standard is to rebalance the relationships among patient rights protection, physicians’ professional autonomy, and the regulatory function of the judicial system within the healthcare system (75). The new standards promulgated by the American Bar Association mark a departure from rigid reliance on customary medical practices, encouraging courts to integrate evidence-based medicine into the adjudication of medical malpractice cases. This restatement offers healthcare practitioners and their affiliated institutions an opportunity to revisit the framework of medical negligence assessment and to more explicitly align their practices with the core objectives of advancing patient safety and optimizing medical services. Understanding the implications of this regulatory adjustment is critical for balancing patient safety, physician autonomy, and the proper role of the legal system in the healthcare ecosystem (76).

Structural imbalance in rights distribution serves as the fundamental structural cause of the contemporary doctor–patient trust crisis (76); furthermore, the legal dilemmas encountered in doctor–patient trust relationships promote the integration of “doctor–patient–trust–based relationship” theory and “doctor–patient community” theory (63). Medical liability for harm is designed to resolve doctor–patient disputes and protect the legitimate rights and interests of both parties by integrating normative logic with practical rationality (46). This one-dimensional interest–protection mindset leads to either the diffusion of responsibilities or the erosion of rights, which ultimately damages the doctor–patient relationship and fosters an atmosphere of mutual vigilance and confrontation. The special doctor–patient relationship constitutes the core of legislation on iatrogenic faults (77), and this relationship forms the foundation for the legal obligations that treat physicians owe to their patients, the basis for establishing medical fault liability, and the prerequisite for delineating the boundaries of physicians’ special legal obligations and liabilities. As the fundamental pillar for physicians to fulfill higher-order professional responsibilities (78), the doctor–patient relationship requires balanced coordination of public and private interests within the doctor–patient legal framework (62). It is therefore necessary to reexamine the issue of iatrogenic injury, seek balanced coordination between private and public interests (62), effectively address structural imbalances in doctor–patient interactions, and promote the development of a harmonious doctor–patient relationship (8).

Within the medical liability system, multiple conflicting and incongruous values must be applied concurrently, and no single value can supersede or override any other value. The core values encompassed by this system include corrective justice, infringement deterrence, victim compensation, patient safety, cost diversification, social welfare maximization, and the provision of accessible and affordable medical services. These values require prioritized application and selection, which eliminates the traditional path dependence stemming from value egalitarianism and simplistic value monism. In the proposed ranking, patient safety holds the highest priority, followed by corrective justice and deterrence, and ultimately distributive benefits and welfare outcomes. No value included in this framework is assigned an absolute priority weight. This value ranking is consistent with the structure of social justice and permeates the entire system of medical tort law, exerting a fundamental influence on the legislation of medical tort liability, judicial interpretation practices, and judicial adjudication by judges.

## The “regulating valve” of medical liability: an elastic framework for establishing factual causation standards

Cross-national variations in the interpretation and enforcement of causal attribution can be substantial, which reflects divergent societal attitudes toward healthcare accountability and patient rights and ultimately affects the extent to which patients hold healthcare providers liable for medical negligence. Medical liability can be conceptualized as a context-dependent social phenomenon whose normative significance, practical impact and actual effects vary across different institutional settings. The uncertainty surrounding the standards for establishing negligence and causal attribution undermines judicial fairness and exacerbates doctor–patient conflicts from the perspective of legal systems. To avoid being found liable for negligence, medical practitioners may prioritize self-protection when they make trade-offs between “compliance with legal requirements” and “life-saving treatment” or adopt defensive medicine when they balance “empirically based clinical diagnosis” and “evidentiary documentation.” In practice, this dynamic replaces the traditional “harm-control harm” practice with a new form of medical infringement. Since the majority of the harm incurred by patients involves damage to life or health, civil compensation neither provides substantive redress for victims’ rights nor can restore the victim’s life and physical condition to their preinjury state. Even the compensation for the psychological harm suffered by victims and their families often remains inadequate under the current framework. Accurate identification of causality in medical harm is among the most critical steps in the resolution of medical disputes (78). Currently, China’s legislation lacks independent provisions on causal relationship determination, resulting in the absence of clear causal identification standards and corresponding liability allocation principles (79). This deficiency impedes the effective resolution of medical harm disputes. Therefore, it is necessary to refine causality identification theory to maintain a balanced status between physicians and patients in medical harm cases.

### Factual causation and legal causation: distinguishing logical causality and mitigating proof transformation

The core prerequisite for medical institutions to assume tort compensation liability is that a causal relationship exists between their medical negligence and the consequent harm sustained by the patient, and the scope of compensation should be strictly limited to the specific damage directly caused by such faults (80). The Tort Liability Chapter of the Civil Code of the People’s Republic of China stipulates that the causal relationship between medical malpractice and patient harm is a core element for medical institutions to bear tort liability when a patient sustains damage. The four constituent elements of tort liability are the illegality of the conduct, the subjective fault of the actor, the occurrence of damage, and the causal connection between the conduct and the harmful consequence. Medical malpractice refers to medical treatment that fails to comply with mandatory and generally accepted professional quality standards and can be defined as a breach of diagnostic and nursing obligations or harmful consequences arising from obvious negligence. Medical damage refers to the adverse outcomes experienced by a patient caused by the fault of medical institutions and their medical personnel during the process of diagnosis and treatment (81), and the establishment of such entities must satisfy the core requirement of a “causal association with medical malpractice” (81). In cases of medical malpractice, the causal relationship between clinical conduct and adverse outcomes must be verified (82), and causal analysis constitutes a core step in medical damage assessment (83). If an adverse clinical outcome still occurs despite the administration of treatment with due diligence in compliance with established medical care standards, no causal link exists between the treatment and the outcome (83). The core issue in medical liability adjudication lies in the identification of causal linkage, and the establishment of liability is contingent on the confirmation of this causal relationship. Causality is an objective prerequisite that precedes the determination of liability: no liability can be established in the absence of a valid causal connection (83).

The allocation of the burden of proof in medical malpractice disputes is strongly correlated with the outcome of judicial rulings. Current Chinese legal provisions reasonably assign the burden of proving the factual causation among the constituent elements of medical damage liability to the patient. However, owing to the imbalance in evidence collection capacity and information accessibility between physicians and patients, this burden of a proof allocation rule may lead to substantive inequality between the two parties to litigation. Patients are confronted with challenges arising from the subjective fault element and the professional requirements for proving causal relationships and face the risk of losing the lawsuit because they fail to meet the burden of proof when they are seeking judicial remedies for their infringed rights and interests (43). Requiring patients to master professional medical and nursing knowledge to substantiate causal facts, without establishing presumptions or lowering the standard of proof, constitutes a misjudgment of patients’ evidential capacity and ultimately leads to substantive inequality between equal parties in civil litigation (43). Pursuant to Article 4 of the Interpretation of the Supreme People’s Court on Several Issues Concerning the Application of Law in the Trial of Cases Involving Medical Damage Liability Disputes, where a plaintiff fails to adduce evidence to establish the causal link between medical conduct and the resultant damage, the patient may apply for medical damage appraisal to prove the existence of causation. “Whether a causal link exists between the diagnostic and therapeutic conduct and the resultant damage” is specified as a core appraisal item, which is designed to remedy the patient’s weak evidential presentation capability. The causes of medical damage are complex and heterogeneous. To a large extent, the determination of whether medical conduct constitutes medical malpractice and whether a causal link exists between medical conduct and injury outcomes hinges on the professional expertise and practical experience of clinical medical experts, and a proper determination can be made only in adherence to the principle of peer review (22). Professionalism and scientific rigor are core requirements for medical malpractice damage assessment.

Causation can be divided into factual causation and legal causation (84). Factual causation in the context of medical injury refers to injuries resulting from medical errors. From a pathophysiological perspective, the probability and magnitude of contributing factors are quantified, and causality is inferred through established probabilistic criteria. From a legal perspective, legal causation is premised on the establishment of factual causal linkage, which further delineates the scope of the defendant’s liability. For the establishment of tort liability, the core question to resolve is whether an objective nexus exists between the harmful act and the infringement of rights—that is, whether the harmful act constitutes a conditio sine qua non for the resultant harm. As a type of forensic medical evidence, expert appraisal opinions analyze and evaluate only the factual causal relationship between the alleged conduct and the resultant injury, as well as the degree of causal contribution, and do not entail determinations of legal causation (85). The causal relationship assessed in medical damage appraisal constitutes factual causation rather than legal causation (37). Within the scope of tort liability, causation refers to the causal connection between a wrongful act and the resulting damage (86), and the core issue to be resolved is the scope of damage. Legal causality primarily serves to determine the scope of liability and delineate the degree of corresponding liability. It requires a comprehensive evaluation based on factual causality and the conclusions of medical appraisal, with relevant factors, including social justice, taken into consideration in accordance with the law (86). In Chinese medical damage litigation proceedings, the standard for proving causality is relatively lenient. Judicial judgments often lack explicit elaboration on factual causation and legal causation, which has led to the perception that causality assessment is not effectively applied in medical tort judgments (37). In judicial judgments, the determination of factual causation mostly relies on the repetition of content from medical appraisals (87). Neither expert testimony nor the judicial finder should bypass factual causation to directly determine the degree of causal contribution.

A forensic medical appraisal opinion constitutes a scientific judgment on the causal association between medical malpractice and the resulting harm, which is formed on the basis of scientific and technological evaluation of this association at the factual level. From a legal perspective, the judgment of legal causality serves as an instrument for value judgment and liability allocation and is not equivalent to factual causality in natural science. Therefore, it does not need to reach the same level of precision and rigor as natural scientific causal judgments do. The judgment of legal causality is subjective: it is a conclusion reached in individual cases that integrates legal norms and value considerations on the basis of ascertained case facts. When determining legal causality, adjudicators never defer to the conclusions of medical expert appraisals. At present, the laws and regulations regulating medical malpractice liability disputes do not suffer from problems such as the failure to clearly distinguish between factual causality and legal causality, nor do they lack relevant provisions on the connection between the two types of causality (88). In practical applications, this state falls within an ambiguous range or contains errors. Article 12 of the Interpretation on the Application of Law in Cases Concerning Medical Malpractice Liability Disputes stipulates that “The determination of causality and the degree of causality set forth in an appraisal conclusion constitutes a legal finding of causality, rather than the factual causality established in general forensic clinical judicial appraisal.” This revision can return forensic appraisal to its proper scope of identifying the factual causal relationships recognized by forensic clinical appraisal (89). In 2005, the Shanghai High People’s Court issued the Guidelines for Handling Medical Malpractice Compensation Dispute Cases, which stipulate that “the determination of causality shall be conducted separately through analytical frameworks for direct causation, equivalent causation, and complex causation, on which the causal contribution of medical malpractice is determined.” The guidance further clarifies the specific adjudication rules: if a “simple causal structure” is directly induced by medical error, it can be adjudicated as direct causation; if damage occurs from the combined effect of multiple causes, it should be determined as a complex causal relationship; if medical malpractice substantially increases the objective likelihood of harm and constitutes an indispensable condition for harm occurrence, it should be adjudicated as adequate causation. In 2008, the Chinese Medical Association also incorporated the concepts of direct causality, indirect causality, and adequate causality into its training manual for technical appraisal experts of medical accidents. In 2010, the Tort Liability Law of the People’s Republic of China was officially implemented, which established the dominant position of adequate causality in China’s medical litigation adjudication (90).

Medical malpractice must be necessary for the resulting damage; the occurrence of medical malpractice and subsequent harm alone does not substantiate the existence of causation. In practical adjudication, proving causal linkage is extraordinarily challenging: establishing causality solely through probabilistic inference is inadequate, and a higher degree of certainty regarding the connection between the physician’s conduct and the adverse outcome is needed, a standard that is practically unattainable in the majority of cases. Shifting the burden of proof from the patient to the physician does not resolve this dilemma, as physicians face equal difficulty in proving that the patient’s death or severe health impairment was not caused by medical fault. In medical damage dispute cases in China, courts tend to issue rulings in favor of patients on the basis of considerations of protecting vulnerable groups and mitigating tensions in doctor–patient relationships. During the trial process, judges rule to provide patients and their families with appropriate financial compensation on the basis of the principle that “it cannot be ruled out that the health damage is unrelated to the medical actions,” thereby reflecting societal compassion and humane care to mitigate medical disputes (91). In some cases, although courts are unable to confirm a causal relationship between medical malpractice and adverse outcomes via forensic appraisal, they still rule out that medical institutions bear partial liability without establishing a valid causal construction; such judgments remain subject to scholarly and practical debate (92). A prominent phenomenon driven by the “result-oriented” adjudicative approach is that judges prematurely introduce subjective value judgments and rigidly establish causal links between substandard medical diagnosis and treatment and the final harm incurred. While this approach facilitates the protection of the rights and interests of vulnerable parties in medical tort disputes, it substantially weakens the evidentiary standard for proving causal relations and erodes the rigor of legal reasoning (16). In judicial practice, cases in which neither the appraisal opinion nor the existence of medical malpractice and its causal relationship have been conclusively determined exist, yet medical institutions are still ruled to bear compensation liability after judicial analysis (86). In Dispute over Medical Damage Liability between Dongpu Lianxi Medical Clinic (Tianhe District, Guangzhou) and Others v. Liu Hongzhou et al. (2018 Yue 01 Min Zhong Civil Judgment No. 595, Guangzhou Intermediate People’s Court), the adjudicating court failed to present a reasonable reasoning on the causal link between medical malpractice and the patient’s death. Instead, the judge evades the review of factual causation by citing a limited understanding of the causal relationship in medical damage liability, which renders the subjective fault of the medical institution the decisive factor for causation determination in this case, ultimately leading to an excessive tilt of the interest balance in favor of the claimant.

In Zhai Mingwu et al. v. Cixi People’s Court regarding Medical Damage Liability Dispute (2013 Zhejiang Yong Zhong Min Civil Judgment No. 186, Ningbo Intermediate People’s Court, Zhejiang Province), the patient was misdiagnosed at the initial consultation, and surgical intervention became infeasible by the time of the second admission. Given that the technical appraisal report had already confirmed that the patient was diagnosed with an advanced high-malignancy tumor, making it difficult to prove the causal relationship between medical negligence and the patient’s death, the court replaced factual causation with legal causation to balance the interests of the medical institution and the patient and ruled out that the medical institution should bear partial liability for compensation for death damage and compensation for mental distress.

The causal relationship between damage and tortious acts is a fundamental principle that must be adhered to in terms of tort liability. Nevertheless, certain judicial bodies have recognized the deficiencies of this doctrinal approach and have thus adopted the framework of “compensation” rather than “reimbursement” (92). The vast majority of medical injury cases involve multiple contributing causes leading to a single adverse outcome, with the contributing factors at minimum including the patient’s underlying health condition and nonstandardized diagnostic and therapeutic practices. The traditional dominant evidence rule is probability-based, which uses overall statistical data to quantify the causal probability for individual patients and establishes a high-probability standard of proof. This framework is rooted primarily in the fact that a fully objective and factually accurate standard of proof cannot be achieved in litigation. Probability constitutes an abstract standard: while percentages such as 51% or 75% serve as reference benchmarks, the probative force of individual evidence items cannot be easily quantified into a specific numerical value. Under the probability-based standard, the difficulty of proving causation in medical malpractice is an inherent flaw of the current proof standard system, which must be addressed by relaxing the proof requirement. Empirical data from 2024 indicate that the court admission rate of expert appraisal opinions reaches 94%, while the proportion of parties filing objections to such opinions is as high as 64.5% (93). Technical appraisals of medical malpractice should focus on identifying and examining the factual causal linkage between negligent conduct and the resulting harm. Both the causal relationship for establishing liability and the causal relationship determining the scope of liability require analysis of the facts to be proven and the applicable standards of proof (36). Establishing causality is a notably complex component of the evidentiary process, as even legally conducted medical interventions cannot consistently guarantee anticipated outcomes (83). This gives rise to the following question regarding the required standard of certainty: is a threshold of probability sufficient, or is a degree of probability equivalent to certainty required? From the patient’s perspective, if the traditional standard for factual causation is applied strictly, they are highly unlikely to experience damage.

### Dilemma and breakthrough in factual causality: the localization of proportional causality theory

Both fault liability and causal attribution incorporate the requirement of foreseeability of “some” unreasonable harm (85). Medical conduct satisfying the elements of the foreseeability rule establishes a causal link between the conduct and the resulting medical harm (80). “Damage” herein refers to legally relevant harm, i.e., damage that is eligible for legal compensation. The determination of causation constitutes a process from quantitative change to qualitative change, which delineates the “scope of doubt between proof and disproof of a fact.” Probabilistic assessment is conducted via observable and quantifiable specific indicators and relies on statistical data to carry out objective quantitative analysis. With respect to the ascertainment of causal relationships, when the probability that a medical error caused the plaintiff’s harm is greater than or equal to 90% and all other potential causes can be reasonably excluded, medical error may be determined as the sole cause of the damage (84). Factual causation is generally established through the “but-for” test. The plaintiff bears the burden of providing a causal nexus between the defendant’s tortious wrongful act and the resultant harm; specifically, in the absence of wrongful conduct, the injury would not have occurred. This framework is designed to quantify the attribution of liability. When the probability of causation is less than 50%, conventional judicial practice tends to lower the probability of finding causation established or even dismiss the claimant’s claim outright. This adjudicative approach overlooks the incremental contribution of each contributing factor to the harm outcome in multiple-cause single-effect scenarios, which readily gives rise to imbalances in liability allocation.

In medical injury cases, the causation scenario is typically characterized by “multiple causes leading to a single outcome”: the patient’s underlying disease progression and the fault of the medical institution jointly contribute to the patient’s harm, which constitutes causal concurrence. To determine causality, it is necessary to clarify not only the binary judgment of “whether causality exists” but also the quantitative judgment of “causal contribution magnitude” (94). Specifically, this includes confirming whether the fault of the medical institution must meet a certain threshold to establish causality and verifying whether the patient’s underlying disease progression is accelerated or even directly leads to death as a result of the institution’s fault. All causal judgments are inherently based on probabilistic possibility (95). Against the backdrop of evidence-based medicine-driven medical development, accurate causal estimation relies primarily on rigorous scientific experimental data (96). The identification of factual causality encounters significant challenges. Grounding causal identification in probability theory renders the process exceptionally complex in special scenarios such as medical injury disputes. This complexity prevents the identification of factual causality from meeting established adjudicative criteria, consequently impeding the effective protection of victims’ legitimate rights and interests. This issue has emerged as a core predicament in judicial adjudication, constituting a prominent challenge to the burden of proof. The doctrine of proportional causation is formulated to address the limitations of the traditional “all-or-nothing” causal rule. Its core logic lies in avoiding entanglement over whether a causal relationship “definitively exists” and instead requiring claimants to prove the probabilistic likelihood of causation between the tortious act and the resultant damage. To date, how to accurately determine the probability and magnitude of medical negligence, as well as how to scientifically and reasonably quantify such probabilistic likelihood, remains an unresolved issue. Medical causality typically exhibits “statistical correlation” rather than “logical necessity” (97), and in medical practice, fault factors rarely reach the standard of being a definitive “cause.” The professional and technical attributes of medical and nursing practices, combined with the complexity of causal relationships and the absence of flexible standardized allocation of the burden of proving causation, collectively lead to a situation where merely granting patients the right to apply for expert appraisal cannot substantively resolve the proof dilemma. Therefore, further exploration is needed to mitigate the burden of proof with respect to the causal element (43), and it is necessary to develop mitigation frameworks for factual determination within the causal elements of medical malpractice (43). Whether the high-probability standard required for causation in traditional tort law is satisfied and whether factual causation in a given case can be established on the basis of a certain probability remain within the discretionary power of the judge. It is necessary for statutory law or judicial interpretations to further clarify the specific rules and applicable conditions for causation determination in medical tort cases and explicitly introduce the doctrine of causal contribution. Specifically, on the basis of evidence-based medical evidence and the court’s preliminary calculation of causal probability, with reference to professional appraisal opinions, this approach breaks through the traditional rules of causation identification and appropriately introduces a relaxed standard for proof of causation, thereby balancing the interests of both physicians and patients and effectively resolving the difficulties associated with determining causation in such cases.

In the scenario of medical damage with multiple causes and one single effect, the ideal causal inference framework requires that the causality between a given factor and the resulting damage is established with a high degree of statistical probability (37). How to achieve high-probability causal identification in the scenario of multiple causes and single effects is still an unresolved problem. The 50% threshold is a concrete instantiation of the high-probability causality criterion, yet it still exhibits arbitrariness and uncertainty when applied to the judgment of multiple-cause single-effect cases (37). In the “all-or-nothing” causal attribution model, statistical data primarily support judges’ reasoning regarding the probability of causation (16). If the probability of the existence of causality is greater than the probability of its nonexistence, causality can be deemed to exist (79), and the standard of proof required must reach the level of probability that excludes reasonable doubt. When the probability of medical error and resultant harm does not exceed 50% under the “no testing” scenario, the standard for proving causation is lower, and the theory of “cumulative causation” is applied. Under this framework, the role of medical error in the occurrence of harm is classified as either a “contributing factor” or a “substantial factor” on the basis of “reasonable medical certainty.” For instance, in the medical malpractice case of Doull v. Foster (2021 Supreme Judicial Court of Massachusetts), the judicial discussion centered on whether to adopt the traditional “but-for” causation standard or the “substantial contributing factor” causation standard. For causal relationships that cannot be established via the but-for causation framework, adjudications can be conducted leveraging existing epidemiological data or evidence-based medicine methodologies, with the threshold of “reasonable medical certainty” (i.e., reasonable probability) applied (84). In the case of Ribeiro v. R. I. Eye Inst., 138 A.3d 761 (R. I. 2016), the court clarified that in the context of medical malpractice litigation, an expert witness’s opinion may only be admitted where it meets the “requisite degree of certainty,” and the expert opinion must attain “reasonable medical certainty—that is, probable certainty” (98).

Causality constitutes the core challenge in medical damage assessment (97). In the practice of compensation, as a perspective on factual causality, the ambiguity of this concept also poses challenges to the alignment between “tort liability” and “damage compensation” (99). The “all-or-nothing” principle creates difficulties in balancing the interests of physicians and patients when medical tort claims are adjudicated. Moreover, the judicial proof methodology has shifted from relying on “highly comprehensive” statistical analysis and corroborating evidence to an evidence chain characterized by definite credibility and probabilistic sufficiency (100). In other words, the lower the probability of damage occurring from a given act is, the weaker the foundation for the proposition that the compensation obligation arising from such damage is rooted in the act itself (101). The legitimacy of the application of probability-based causal inference lies in the adoption of an “all-or-nothing” threshold mechanism in this framework: the proof of compensation claims must satisfy a specified probability threshold. Below this threshold, no liability is established (corresponding to “nothing”); above this threshold, full liability is recognized (corresponding to “all”) (85). Owing to the complexity of human physiological functions and the inherent nature of diseases, all clinical practice guidelines and expert consensuses are applicable to only approximately 80% of patients (102). Applying probability as the statutory criterion for ascertaining the causal relationship between medical practices and resulting damage to medical damage liability adjudication is unreasonably stringent and fundamentally erodes the foundational premise for the establishment of medical liability insurance systems. Naturally, there are divergent perspectives on this issue, with some scholars arguing that a standard of proof characterized by high probability should be maintained (36). The factual causation established by the “all-or-nothing rule” and “reasonable medical certainty” share a common core: both are based on the probability of adverse harm outcomes and differ only in their applicable standards of proof and applicable factual scenarios (84).

On the basis of a given probability, the existence of causation is excluded in certain extremely low-probability and unpredictable scenarios, thereby exempting the medical party from liability for damage resulting from specific special causes. Proportional causation is applied to determine the probabilistic association and proportional relationship between harmful factors and specific injury outcomes (103). In the field of comparative law, probabilistic causality theory is introduced primarily at the liability establishment stage to construct proportional liability theory (104). Proportional causality is an outcome of the evolution of legal thinking from determinism to flexibility, and it enjoys a substantial degree of acceptance in judicial practice. As a complex discipline, medicine frequently involves multiple contributing causes that jointly lead to adverse injury outcomes. To establish legal liability, it suffices to prove that the tortious act in question is capable of increasing the probability of the outcome’s occurrence (79). By screening out statistically significant factors associated with outcomes, this approach demonstrates the correlation between suspicious factors and the observed results. In Li v. A Hospital (Medical Damage Liability Dispute; 2019 Su 04 Min Zhong No. 3593, Civil Judgment of Changzhou Intermediate People’s Court of Jiangsu Province), the judicial appraisal, conducted in accordance with the 2014 Chinese Expert Consensus on Diagnosis and Treatment of Coronary Artery Calcified Lesions, identified coronary rotational atherectomy as an optional surgical technique. Domestic and international academic literature indicates that postoperative malignant thrombosis occurs at an incidence of 1–3.1‰. On the basis of these data, the appraisal concluded that there was no causal association between the medical intervention and the patient’s death, a finding subsequently affirmed by the court. In this case, the theoretical incidence of postsurgical complications was applied to exclude the causal relationship between medical conduct and patient death; specifically, the causal association was ruled out on the basis of the low incidence of fatal complications reported in the literature.

However, determining causality through probability-based approaches also has inherent flaws. Specifically, when the legal protection of core interests is at stake, the goal of capping liability may ultimately result in causal attributions being incorrectly rejected (105). The establishment of causal attribution is grounded in probabilistic reasoning. If the probability that the defendant’s conduct caused the alleged damage reaches 30%, factual causation is deemed established. However, there remains a 70% probability that the defendant’s conduct did not cause harm. If no actual causal linkage exists between the conduct and the damage, how can holding the defendant liable for compensation be considered consistent with the principle of justice? (36), This finding demonstrates that the law has adopted the necessary adjustments to deliver fair adjudications in response to the complexity of modern medicine and the inherent limitations of medical traceability technology (42). Currently, causality determination no longer focuses on whether the probability threshold is above or below 50%. Instead, it infers factual causality on the basis of a certain degree of probability. Proportional causality theory identifies causal links by assessing the likelihood that a tortious act caused the resulting damage and calculates the tortfeasor’s liability proportion in accordance with this probability. A factual causal framework constructed on probability with a fixed degree threshold is more reasonable than the traditional probabilistic proof standard. Considering the particularity of medical damage liability, adopting a certain probability threshold (30%) or proportional causality theory to establish a relaxed standard of proof for fault and causal relationships constitutes an effective approach to resolving medical and nursing damage disputes and achieving substantive equality among nurses, physicians and patients.

### From attribution to liability: functional reconstruction of causative potency theory in proportional compensation for medical damages

In medical appraisal, the causal attribute of liability for medical practices is determined primarily by two factors: whether the medical provider has committed medical malpractice and whether a causal association exists between the aforementioned malpractice and the patient’s injury (106). Common expressions in appraisal opinions include the terms “degree of participation,” “degree of fault involvement” and “degree of responsibility involvement.” In Chinese civil law, the concept of “causal power” is typically adopted to describe the degree of participation, which refers to the extent of a specific cause’s influence on the occurrence or expansion of harmful results among multiple contributing factors. The Interpretation on Medical Damage Disputes divides causality into six categories: total, major, equivalent, secondary, minor, and none. In judicial practice, the vast majority of appraisal opinions define causal power within a single categorical range. The normative provisions on causality and fault in the Judicial Appraisal Guidelines stipulate the following: for causality, the classifications are no causal relationship, probable causal relationship, and confirmed causal relationship; for fault, the degrees are minor fault, secondary fault, equivalent fault, major fault, and total fault. Current standards and specifications for judicial appraisal are overly broad and ambiguous and lack unified uniform norms, which leads to substantial divergence in practical application (107). Constrained by the current level of science and technology, it is difficult for medicine, statistics and forensic identification to provide accurate quantification of the strength of causative potency, and there is no mature overseas experience available for reference. The Restatement of Tort Law III: Apportionment of Liability enumerates specific factors to be considered when apportioning liability, including “the degree of contribution of the causal connection, the sequence in which the conduct of multiple parties caused the harm, and the comparison between the risk created by the conduct and the actual harm suffered by the plaintiff” (23); however, these limited reference factors remain highly ambiguous.

The specific allocation of causative potency falls within the scope of liability and legal causation (36). Establishing factual causation in identification serves as the prerequisite and foundation for apportioning proportional causal responsibility. Medical technical faults are the primary factor influencing the determination of causal attribution (17), and the degree of fault and causal attribution contained in the appraisal opinion constitutes only a technical judgment. When translated into legal concepts, it refers to the contributory proportion of faulty conduct within the established causal framework (86). Causal potency does not contradict the principle of full compensation in tort damage; however, the doctrine of full compensation does not require identical compensation for all losses. The term “full” in this context refers to requiring the medical practitioner to compensate for all damage falling within the scope of their fault. In the adjudication of the majority of medical disputes, judges allocate proportional liability to medical institutions on the basis of the degree of fault in the diagnosis and treatment process or the causal contribution of medical conduct to adverse outcomes, and these two criteria are logically equivalent. Therefore, the application of causative potency to mitigate liability is legally justified when the scope of liability is defined and legal causation is ascertained (36). The doctrine of causation enables the allocation of liability in cases where harm results from multiple causes. When medical risks and uncertainties are confronted, it facilitates the implementation of the principle of self-responsibility, advances the balance of interests, and upholds fairness and justice.

“Causative potency” and “factual causation” are two distinct concepts that differ in terms of institutional attributes, operational principles, and normative functions. From the perspective of institutional attributes, causative potency essentially constitutes a standard of proof, whereas factual causation serves as a method for responsibility allocation. In terms of operational principles, some courts in Chinese judicial practice combine the causal relationship required to establish liability with the causal relationship determining the scope of liability. A prominent issue in medical tort adjudication lies in the application of causal rules: the erroneous conflation of these two types of causal judgment frequently occurs. Specifically, the application of rules concerning medical malpractice contribution degrees or causal participation ratios should be premised on the prior establishment of factual causation as the foundation for attribution (108). Prior to applying the causative potency rule, a preliminary determination of factual causation should be conducted (105). In essence, the proportionality of causation only takes effect when a factual causation is established, which then influences the extent of compensation liability. If no factual causation exists, no assessment of causative potency is needed (37). Owing to the challenges in determining causality in medical tort facts, judicial trials still tend to evade this premise through conventional causative potency rules. When it is difficult to confirm the existence of a factual causation, liability is directly allocated on the basis of the proportional contribution of the medical malpractice factor to the resulting harm; this approach bypasses the conventional causal reasoning requirement and directly establishes the attribution of liability. From the perspective of the normative function, the core role of establishing causation in liability adjudication is to limit overly extended causal chains and balance the allocation of liabilities between parties. Nevertheless, the application of the aforementioned proportional allocation method typically leaves medical practitioners disproportionately exposed to undue liability.

Analyzing the causal relationship between medical malpractice and resulting damage and assessing the causal attribution of medical errors to damage outcomes constitute both a core focus and a major challenge in medical damage appraisal (84). Current approaches to addressing ambiguous causal relationships in judicial proceedings have resulted in the improper application of the concept of contributory medical negligence (108). Expressions in judicial judgments such as “the causal link with the damage outcome cannot be completely excluded” and “causality exists to a certain degree” reflect the ambiguity of factual causation, which creates difficulties for the determination of tort liability under such circumstances and provides no valid basis for the application of causal contribution. In the medical damage liability dispute heard by the Supreme People’s Court of China (Case No. 55, Min Shen Zi 2013, Supreme People’s Court), the court upheld the judgment requiring the hospital to bear damage liability in a certain proportion on the basis of the concept of “causal contribution.” The patient’s death resulted from secondary systemic multiple organ failure caused by sepsis, and the outcome was associated mainly with the patient’s individual constitution and preexisting underlying disease. Nevertheless, the medical conduct in question was faulty, and it maintained a certain causal link with the patient’s death; hence, the responsible party should bear secondary liability. When courts apply causal contribution in the context of unclear causality, such practice constitutes an abuse of the causal contribution framework and excessively prioritizes the interests of patients. While patients who sustain harm during the course of diagnosis and treatment are entitled to reasonable remedies, unjust treatment against medical institutions must be avoided. The introduction of fault contribution in medical damage liability adjudication is designed to define the scope of tortfeasors’ liability more reasonably, and it should not be utilized by courts as a tool to infinitely expand the liability of medical institutions; as such, the practice deviates from the original intent of the causal contribution doctrine (108).

The causative potency determined by appraisal opinions plays a critical role in judicial adjudication. It fulfills the policy requirement of mitigating doctor–patient conflicts and facilitates the establishment of standardized loss-sharing rules (37); it is a wise move to balance the interests of both doctors and patients under the Chinese legal framework (37). The causative potency rule is derived from Japan’s concept of the “degree of contribution.” It aims to quantify the causal influence of a cause on its result by evaluating the extent to which a specific causal factor among multiple contributing causes affects the occurrence or aggravation of the result. The application of this rule is premised on the confirmation of causality (109). The causative potency ratio fails to reflect the underlying medical occurrence probability between medical errors and adverse harm outcomes and indicates only the relative association of the event with other common harmful factors (16). However, judges and judicial appraisers frequently confuse causality with the probability of harm and directly adopt proportional liability in adjudication. This practice subverts the traditional causal identification system, undermines the role of causality as an “attribution filter,” and negatively affects the coordination and stability of the tort liability system.

Causal potency theory is pivotal in the domain of medical liability law in China. It was originally proposed to address “multiple causes, single effect” medical tort disputes, allocate liabilities appropriately between physicians and patients, and ultimately achieve a substantive balance of interests between the two parties. Determining liability on the basis of the proportionality of causal contribution is the logical inference and natural outcome of the proportional causality doctrine, which constitutes the conclusion of this theoretical framework and holds great significance for reasonable liability allocation and interest balancing (103). In this context, constructing proportional liability via proportional causation theory and general causation principles and integrating these frameworks at the level of legal causation to reshape the function of proportional compensation in medical damage liability carry substantial research value and theoretical innovation.

## Redistribution and risk sharing of medical tort damage liability under the framework of justice

The constituent elements of medical damage compensation liability need to break through the “compensation-only theory” framework within the liability structure. Grounded in the theory of justice, this paper comprehensively accounts for the nature of the doctor–patient relationship, the characteristics of medical damage, and the core objectives of the medical system and reconstructs the interest allocation scheme of medical damage compensation liability from the perspective of justice theory. This reconstruction achieves a diversified and effective balance between patients’ rights and social public interests in the allocation of medical damage compensation liability, aligns with the international trend of medical damage liability allocation, and highlights the transformation of China’s medical liability adjudication paradigm from “result-based liability determination” to “multifactor joint liability determination” (55). This framework of liability allocation embodies a rational interpretation of medical uncertainty and establishes a prudent balance aligned with the principle of nonmaleficence, incentivizing medical institutions to reinforce their ethical obligations for risk prevention and governance. The theory of justice redirects the focus of identifying medical malpractice from mere technical adjudication to substantive attention to the interests of both physicians and patients. This shift is highly important for advancing the realization of livelihood security objectives and facilitating the regulated development of national healthcare systems.

### Infringement liability regime in the context of medical innovation: normative adaptation and paradigm shift

Liability for medical damage compensation does not exist in isolation; it is intimately closely connected with a range of factors, including a country’s doctor–patient relationship, the operation of the medical system, and the legal environment. The institutional design of such liability should balance the neutrality of the medical discipline and the values of jurisprudence, integrate the connotation of justice theory with the unification of the interests of both physicians and patients in medical damage disputes, and provide a systematic examination of the existing constituent structure of medical damage liability. The loss-sharing rules that have been formed and applied on a long-term basis in China’s judicial practice of medical damage are legally justified: essentially, they adopt “lowering the identification criteria for causation and fault, and adjudicating partial liability” as a strategy to alleviate doctor–patient conflicts (37). “Doctor–patient justice” refers to the coordination of multiple conflicts of interest and rights between physicians and patients and the establishment of a dynamic balance among risk, freedom, safety, responsibility, and rights protection.

The current medical dispute resolution mechanism is grounded in “fault-centered” theory, with the “compensation theory” of damage remedies serving as its core foundation. Owing to the absence of a high-level systemic perspective in its institutional design, this mechanism tends to trigger retributive doctor–patient confrontation, as it prioritizes punishment as the sole approach to dispute resolution. The proliferation of medical disputes and the prevalence of medical violence have triggered an institutional crisis that may lead to the breakdown of the healthcare system, resulting in a lose–lose outcome for physicians, patients, and the broader society (10). A physician’s clinical practice is not only an unrestricted privilege but also a weighty legal and professional obligation. In accordance with the requirements of fault liability theory, damage predicated on faults must be determined by establishing legal causation. The identification of fault and causation in medical damage compensation liability is uniquely complex and requires both the assessment of clinical facts and formal legal evaluation. To evade liability, physicians have an inherent incentive to conceal their negligent conduct. This not only creates procedural barriers to establishing liability in tort litigation but also exposes patients to continued risk stemming from the retention of substandard clinical practices. The U. S. medical malpractice system has long been criticized for failing to fulfill the two core objectives of tort law: compensating injured victims and deterring negligent conduct. For victims of medical malpractice, “the medical malpractice liability system generally delivers very low levels of compensation; even when compensation is awarded on rare occasions, the process is inefficient and disproportionately costly.” With respect to its deterrent effect, empirical research has shown that “a higher perceived risk of medical malpractice liability is not correlated with improved clinical quality” (23). Notably, the negligence liability rule is more efficient than strict liability and can reduce the absolute deviation from the optimal level of precaution (110).

The legitimate function of medical damage compensation liability should be established on the basis of core factors, including the risk, fault, and unpredictability of medical practices. This liability mechanism is designed to guide medical practitioners in making independent clinical decisions and ultimately achieve the coordinated development of interests among medical institutions, patients, the medical industry and the public. It is necessary to realize the fairness of liability allocation from the integrated perspectives of medicine, law and sociology and integrate the concept of justice into both medical and legal domains to coordinate and resolve conflicts arising in medical practice. This constitutes the inherent essence of justice in medical damage compensation liability.

Conflicts of interest among patients, the medical industry and the public interest reflect deeper-seated social factors underlying the doctor–patient relationship and play a decisive role in shaping the evolution of such relationships. The essence of doctor–patient justice is social justice: it represents a contemporary evaluation of medical damage compensation liability on the basis of the value dimensions and standards of justice and enables the transition from individual-oriented justice to socially oriented justice. If the institutional design of medical damage compensation liability focuses only on damage completion while neglecting the tension between clinical decision-making freedom and damage compensation, it will intensify only existing doctor–patient conflicts and lead to an imbalance in overall social interests. The overexpansion of liability borne by the medical industry will eventually impose fragmented burdens on the whole society; moreover, adhering only to the principle of individual justice will generate unnecessary legal and moral costs. Therefore, the core goal of the medical damage compensation liability system should shift from the traditional “harm offsetting” approach to “balancing liability and harm” (10). A balance between harm and liability should be struck to achieve substantive equality between the rights and interests of patients and the responsibilities of medical providers. To prevent liability rules or the medical injury compensation system from being excessively biased toward any party, we need to ensure that the medical damage compensation system meets the reasonable expectations of patients and gains acceptance from the medical industry and the public, steers the system toward fairness and justice, and protects the legitimate rights and interests of both patients and medical providers (111). Accordingly, safeguarding the legitimate rights and interests of both parties has been established as the core guiding principle of legislation, which provides a clear direction for subsequent legislative activities (112). The design of medical damage compensation liability should be centered on patients’ rights, with legislative norms tilted in favor of patients. However, such a tilt should not be arbitrary or unlimited; rather, it should be implemented on a conditional basis, and a dedicated mechanism should be established to align medical innovation with liability rules to construct a justified institutional framework.

### Risk-sharing mechanism: the transmission pathway of medical liability insurance for resolving doctor–patient conflicts

With increasing public awareness of rights and interests, the issue of medical risk mitigation has become an unavoidable concern. Factors such as the limitations of current medical technology and the inherent complexity of medical activities make it impossible to fully eliminate medical risks and frequently contribute to the occurrence of medical disputes. Against this backdrop, balancing the interests of both medical practitioners and patients has become an intractable dilemma, with safeguarding patients’ right to compensate and control healthcare institutions’ operational costs (48). The current medical tort liability system fails to respond promptly and effectively to the demands of victims of infringement, leading to prominent practical challenges. Medical risk sharing is established on the basis of balancing the interests of both physicians and patients; hence, it is necessary to introduce social forces to diversify medical risk burdens (48). Medical institutions fundamentally resolve the scenario in which their assets are insufficient to compensate victims, thereby attaining the objective of protecting the legitimate rights and interests of victims who sustain losses from insurance accidents (113). Compared with medical tort liability, medical malpractice liability insurance can promptly and effectively compensate patients for their damage. The current high operational costs of the healthcare liability system, as well as the additional expenditures generated by defensive medicine, have exerted a significant negative effect on medical institutions. To address the multiple risks arising from the uncertainty of medical practice and mitigate doctor–patient conflicts, establishing a sound medical liability insurance system is a critically important institutional solution.

Medical liability insurance is recognized as a critical instrument for mitigating doctor–patient conflicts, diversifying medical risks, and facilitating the stable development of the healthcare industry. In 2018, China enacted the “Regulations on the Prevention and Handling of Medical Disputes,” which introduced guiding provisions for the medical liability insurance system. Article 7 of the Regulations explicitly stipulates that “Medical Institutions are encouraged to purchase medical liability insurance, and patients are encouraged to purchase medical accident insurance.” This achievement established the legislative regulation of medical risk-sharing mechanisms at the legislative level through administrative regulations. The “Basic Medical and Health Promotion Law of the People’s Republic of China,” implemented in June 2020, elevated the medical risk-sharing mechanism to the statutory level, marking the preliminary establishment of a standardized medical risk-sharing framework in China (112). Currently, China has established a medical insurance product and service system, where medical liability insurance serves as the core component, medical accident insurance acts as a complementary component, and multiple other types of insurance develop in synergy (20). However, medical liability insurance is confronted with a “dual chilling dilemma,” characterized by a low willingness to participate among both policy applicants and underwriters across different regions, and its functions of third-party compensation and socialized risk sharing in medical dispute resolution remain to be strengthened (20). The current medical liability insurance system adopts a commercial insurance organizational structure and a voluntary insurance implementation model. Since medical liability insurance has not yet been incorporated into the scope of compulsory statutory insurance, insurance indemnities have not been organically integrated with medical dispute resolution mechanisms, leading to low participation enthusiasm among medical institutions (48). Current development is confronted with bottlenecks, including ambiguous business insurance models, incomplete medical dispute prevention and resolution systems, limited product supply, and insufficient consumer willingness to purchase such insurance products (114). The claims settlement process of medical liability insurance is highly complex and involves multiple professional assessments and legal procedures. The payout process is typically protracted, leading to suboptimal risk transfer outcomes (115).

Although medical liability insurance has been implemented in China for more than 30 years, owing to imperfect legal and regulatory frameworks, the insurance coverage rate remains volatile. This system fails to safeguard the legitimate rights and interests of affected patients and cannot fundamentally improve the doctor–patient relationship (113). To safeguard the legitimate rights and interests of injured parties and diversify medical risk exposure, the implementation of a compulsory medical liability insurance system in China is both an urgent and important priority. This system fulfills an irreplaceable function and aligns with the public interest principle, rendering it a necessary institutional foundation for fostering harmonious doctor–patient relationships. The core objective of compulsory medical liability insurance is to leverage risk socialization to achieve wealth redistribution toward vulnerable groups, thereby realizing distributive justice (116). To prevent such actors from pursuing self-interest at the expense of victims’ interests and triggering doctor–patient conflicts, the legitimate rights and interests of victims should be protected. Through mandatory stipulations on insurance coverage requirements, compulsory underwriting, premium rate determination, and direct third-party claims, compulsory medical liability insurance protects patients’ legitimate rights and interests. Although compulsory medical liability insurance limits the freedom of contract for medical institutions, it does not result in a substantial imbalance of interest when it is weighed against the public interests of protecting the uncertain majority of victimized patients and advancing the safety of medical practices. Admittedly, premium costs for compulsory medical malpractice liability insurance are borne by insured physicians or medical institutions, and such related costs are passed on to patients to varying degrees, which inevitably negatively affects the affordability of healthcare services.

Theoretically, doctor–patient conflicts themselves can be conceptualized as a type of risk. The introduction of corresponding medical liability insurance can improve the completeness of the medical market, alleviate the pressure of damage compensation faced by medical institutions, and enhance mutual trust between doctors and patients, thereby effectively resolving such conflicts. Medical liability insurance functions by clarifying the causal relationships and transmission mechanisms underlying doctor–patient conflicts. Defensive medical practices are strongly correlated with overall treatment costs. This paper verifies that the transmission mechanism through which compulsory medical liability insurance reduces per capita medical expenditure by curbing the defensive medical behaviors of physicians is valid (19). The “economic damage compensation risk dispersion effect” and “doctor–patient conflict mitigation effect” of medical liability insurance can reduce liability pressure resulting from medical accidents, thereby contributing to a reduction in physicians’ defensive medical practices (117). The introduction of compulsory medical liability insurance can improve both the efficiency and fairness of medical dispute resolution. The results of this study reveal that the implementation of compulsory medical liability insurance significantly reduces the probability of medical disputes, which helps alleviate doctor–patient conflicts and facilitates social harmony (19).

The implementation of compulsory medical liability insurance can reduce the premium of medical liability insurance, increase accessibility for medical institutions and practitioners, and guarantee the fulfillment of court judgments or settlement agreements on medical damage compensation (118). South Korea’s medical liability insurance system comprises two core components: commercial medical liability insurance underwritten by private insurance carriers and medical compensation mutual aid operated by the Korea Medical Association Medical Mutual Aid Association. This system has played a critical role in mitigating the growing volume of medical disputes in South Korea and safeguarding the legitimate rights of dispute victims (119). Scholars have also proposed that the French model—which retains the fault liability principle for medical and health damage compensation, preserves the social safety net function of the social security system, and grants state compensation to victims of medical and health accidents that meet statutory requirements such as the specified injury severity—constitutes a risk socialization mechanism worthy of academic reference (120). To address the multiple risks arising from uncertainty in clinical practice and mitigate doctor–patient conflicts, the compulsory medical liability insurance system constitutes a critically important institutional arrangement (113). Medical tort liability law cannot fully realize distributive justice, whereas compulsory medical liability insurance can leverage risk socialization to achieve this goal (113). The intervention and popularization of medical liability insurance can achieve risk diversification, eliminating the necessity of overreliance on biased protection mechanisms in judicial procedures such as the reversal of the burden of proof for patient protection (53). The objective of the healthcare system has evolved from merely enhancing clinical standards to prioritizing both the accessibility and quality of healthcare services.

## Conclusion

As global healthcare systems are subjected to increasingly rigorous scrutiny regarding care quality and patient outcomes, the allocation of liability for medical damage encounters social justice dilemmas and practical challenges. Social justice facilitates the improvement of accountability mechanisms and capacity building for healthcare institutions, strengthens patient safety, improves physician–patient relationships, and optimizes health outcomes. Amid the continuous advancement of medical technology and the growing expectations of patients, the evolution and development of medical liability have become central to advancing social justice. It is therefore necessary to investigate how legal liability addresses issues such as medical negligence, causal attribution, clinical standards, patient safety, and improvement in cure rates. Among these, the criteria for establishing factual causation between medical malpractice and patient harm constitute the core element and “regulator” of medical damage liability. Both the criteria and the theoretical frameworks for determining factual causation require sustained research and iterative reform. The distinct causal methodologies adopted by courts and legislatures to delineate medical liability directly influence the efficacy of patients’ claims for damage and shape an institutional environment conducive to medical innovation.

The causative potency rule (i.e., the degree of participation in medical malpractice) established in Chinese legislation and judicial practice plays a significant and legitimate role in shaping the medical liability system. This rule falls within the legal scope of causal attribution for liability, which is premised on the establishment of factual causation and constitutes the core principle governing the liability relationship between patients and medical service providers. The application of this attenuated causal determination rule, which establishes a standard for factual causation on the basis of a certain degree of probability or proportionality, is highly important for the realization of social justice. Accordingly, at the level of legal causation, proportional causation theory, which generates proportional liability, can be integrated with the causative potency rule to reshape the function of proportional compensatory liability in medical damage cases. The core path to resolving the social justice dilemma of liability allocation in medical damage disputes lies in dynamic system theory based on element ordering. Through this theory, we need to achieve a dynamic balance between patient compensation relief, medical practice autonomy and social operating costs; strike a balance among efficiency, safety, medical innovation and liability assumption; and reconcile the protection of patients’ rights and interests with the incentive for medical institutions and practitioners to maintain high-quality care; and resolve doctor–patient conflicts with the support of medical liability insurance to realize the mechanism of liability transmission and social risk diversification. In today’s complex healthcare environment, it is becoming increasingly important to construct a liability allocation system for medical damage that both upholds social justice and promotes the development of medical practice and to establish a standardized framework of “medical justice.”

## Data Availability

The original contributions presented in the study are included in the article/supplementary material, further inquiries can be directed to the corresponding author.
